# A Surfeit

**Published:** 1893-05

**Authors:** 


### A SURFEIT.

A surfeit in man is called founder in a horse, and is over-eating—eating more than the stomach can possibly convert into healthful blood. Wise men and careful men will sometimes inadvertently eat too much, known by a feeling of fulness, of unrest, of a discomfort which pervades the whole man. Under such circumstances, we want to do something for relief. Some eat a pickle, others swallow a little vinegar, a large number drink brandy. We have swallowed too much, the system is oppressed, and nature rebels; instinct comes to the rescue and takes away all appetite, to prevent our adding to the burden by a morsel or a drop.

The very safest, surest and least hurtful remedy is to walk briskly in the open air, rain or shine, sun or hail, until there is a very slight moisture on the skin, then regulate the gait so as to keep the perspiration at that point until entire relief is afforded, indicated by a general abatement of the discomfort. But as a violence has been offered to the stomach, and it has been wearied with the extra burden imposed upon it, the next regular meal should be omitted altogether.

Such a course will prevent many a sick hour, many a cramp, colic, many a fatal diarrhœa.

